# Factors Associated with Recurrent Ulcers in Patients with Gastric Surgery after More Than 15 Years: A Cross-Sectional Single-Center Study

**DOI:** 10.1155/2018/8319481

**Published:** 2018-11-04

**Authors:** Monica Pantea, Anca Negovan, Claudia Banescu, Simona Bataga, Radu Neagoe, Simona Mocan, Mihaela Iancu

**Affiliations:** ^1^Clinical Science-Internal Medicine, University of Medicine and Pharmacy, Gheorghe Marinescu 38, Tirgu Mureș, 540139 Mures, Romania; ^2^Center for Advanced Medical and Pharmaceutical Research, University of Medicine and Pharmacy, Gheorghe Marinescu 38, Tirgu Mureș, 540139 Mures, Romania; ^3^Surgical Science, University of Medicine and Pharmacy, Gheorghe Marinescu 38, Tirgu Mureș, 540139 Mures, Romania; ^4^Pathological Department, Emergency County Hospital, Gheorghe Marinescu 50, Tirgu Mures, 540136 Mures, Romania; ^5^Department of Medical Informatics and Biostatistics, University of Medicine and Pharmacy “Iuliu Hațieganu”, Louis Pasteur St., No. 6, 400349 Cluj-Napoca, Romania

## Abstract

**Aim:**

We aimed to establish the independent predictive factors (from *Helicobacter pylori* infection, biliary reflux, histologic features of the gastric mucosa, drugs, comorbidities, and social habits) for gastric stump ulcer occurrence more than 15 years after surgery.

**Methods:**

76 patients with previous gastric surgery were included: 21 patients with gastric ulcer (marginal ulcer or ulcer of the rest of the gastric remnant—study group) and 55 controls (nonulcer group).

**Results:**

*Helicobacter pylori* infection tended to be higher in the control group than in the ulcer group (14.5% vs. 4.8%, *p* = 0.43), without statistical significance. Alcohol consumption had a significant positive association with ulcer (*p* = 0.008), while smoking (*p* = 0.064), low-dose aspirin (*p* = 0.063), and biliary reflux (*p* = 0.106) had a tendency toward statistical signification for positive association. On univariate analysis, smoking (*p* = 0.048, OR = 3.15, 95% CI: 1.01–9.93) and low-dose aspirin consumption (*p* = 0.067, OR = 2.63, 95% CI: 0.95–7.68) were significantly associated with ulcer. According to the multivariable regression model, alcohol consumption (OR = 6.68, 95% CI: 1.29–41.14) and biliary reflux (OR = 6.12, 95% CI: 1.36–38.26) remained significantly associated with increased odds of stump ulcer.

**Conclusion:**

Biliary reflux and alcohol consumption, but not *Helicobacter pylori* infection or gastrotoxic drug, seem to be the most important predictors for ulcer recurrence in patients with gastric surgery for peptic ulcer after more than 15 years.

## 1. Introduction

Peptic ulcer disease (PUD) represents a common pathology in gastroenterology practice, affecting 4 million people each year worldwide, with complications reported in 10–20% [1, 2]. The annual incidence of PUD ranges between 0.10% and 0.19% with a significant decline in the last decades [3], due to several therapeutic improvements such as the use of proton pomp inhibitors (PPI) and eradication therapy for *Helicobacter pylori* (*H. pylori*). Improved medical therapy and advanced therapeutic endoscopic techniques steadily decreased the need to perform surgical treatment on PUD patients over the last 20 years [4].

The most frequent endoscopic lesions after gastric surgery are marginal ulcers (MU), which occur on the anastomotic area with a frequency range from 0.6% to 16% [5–8]. The etiology of MU is still unclear, even if several factors have been studied, such as the role of *H. pylori* infection, the importance of surgical anastomosis, and other associated risk factors (biliary reflux) [9–11].

Studies of risk factors influencing the occurrence of complications in gastric stump (ulcers, cancers) are of clinical interest, with the increasing number of gastric surgeries in obese patients or for early gastric cancer [12–14]. The known risk factors for gastric ulcer and bleeding (*H. pylori* infection, gastrotoxic medication) should be questioned in patients with gastric resection to identify predisposing factors that can be different from those in the normal stomach [15]. Therefore, information about the specific features of histologic and endoscopic lesions in the gastric remnant will help to identify the appropriate surveillance and treatment strategies.

Albeit there is no published data available regarding the prevalence of resected gastric patients, the increased incidence of gastric surgery in the past, especially due to the high prevalence of *H. pylori* in Eastern European countries [1], suggests a relatively high prevalence of patients with gastric stump after PUD surgery in our population, with no defined predisposing factors for recurrent ulcer. With more than 10–20 years having passed from their surgeries and many of them having chronic diseases and treatments, they represent a population with a high risk for ulcer and bleeding in the gastric remnant.

The aim of the present work is to establish the independent predictive factors like *H. pylori* infection, biliary reflux, histologic features of the gastric mucosa, gastrotoxic treatment (nonsteroidal anti-inflammatory drugs (NSAIDs), antiplatelet therapy), comorbidities, and social habits for gastric stump ulcer occurrence.

## 2. Materials and Methods

**Figure 1:**
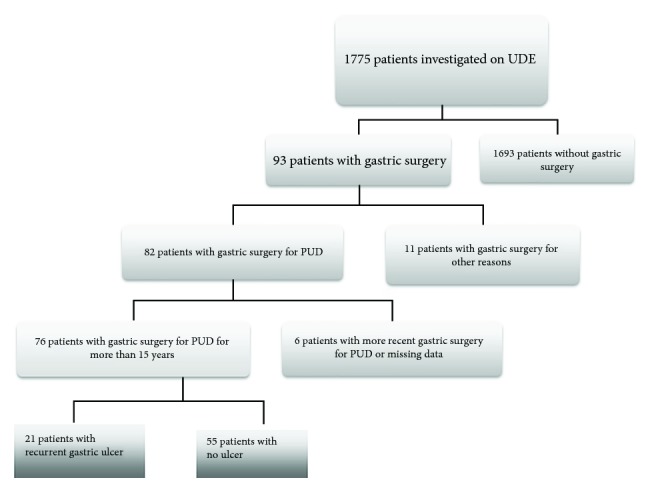
Flowchart of patient selection.

This is a cross-sectional study that investigates in the same time the possible predictive factors and disease occurrence. From a total of 1755 patients who underwent upper digestive endoscopy at the 3rd Medical Clinic in Tîrgu Mureș, Romania, between 2012 and 2015, 76 patients who presented with a history of gastric surgery for PUD of more than 15 years were prospectively recruited using an interview (Figure 1). No data regarding vagotomy was obtained due to the lack of medical records after more than 15 years.

Upper digestive endoscopy (UDE) was performed in all 76 patients with gastric surgery, and according to the presence of ulcers, they were divided into study (ulcer group) and control groups (nonulcer group). UDE was performed for digestive symptoms and anemia, for screening for bleeding risk before starting antithrombotic therapy or having major cardiovascular surgery, or for regular gastric stump complication (every 5 years). A written consent was obtained from every patient included in the study.

The ethical committee of the University of Medicine and Pharmacy of Tîrgu Mureș, Romania, approved this study.

### 2.1. Data Collection

Clinical and demographical data were collected from each patient. We registered the symptoms (heartburn, abdominal pain, vomiting, and nausea) or the presence of anemia in every patient. In order to investigate drug exposure, we used a structured interview and medical records. We recorded treatment with NSAIDs (ibuprofenum, diclofenacum, indometacinum, ketoprofenum, etc., as regular doses within two weeks prior to endoscopy) and antithrombotic therapy (low-dose aspirin (LDA) (75–125 mg/day) (available doses in Romania) and clopidogrelum (75 mg/day)), as well as anticoagulant therapy with acenocumarolum and low-weight molecular heparin (LWMH), at least 2 weeks before performing endoscopy. The reasons for antithrombotic therapies (clopidogrelum, LDA, and anticoagulants) were primary or secondary cardiovascular prevention for ischemic cardiac diseases or arrhythmias (with or without cardiac heart failure), cerebrovascular diseases, and peripheral arterial diseases. Regarding the medical therapy before surgery, none of the patients mentioned the treatment with PPI or *H. pylori* eradication therapy on interview. Some of them admitted having used other antiulcerogenic drugs (bismuth, magnesium or calcium carbonate, aluminum hydroxide, etc.). We used the medical records to check for medical prescriptions and to identify comorbidities (hypertension, ischemic and valvular heart disease, cerebrovascular disease, renal disease, liver disease, osteoarthritis, and diabetes mellitus). Patients with a decreased glomerular filtration rate (GFR) of less than 60 ml/min/1.73 m^2^ were considered the chronic kidney disease group. Patients diagnosed on the basis of clinical and radiographic evidence as having a degenerative disorder of articular cartilage were included in the osteoarthritis group. We excluded patients with gastric surgery for other reasons (gastric cancer, bariatric surgery), patients with a gastric surgery within the last 15 years, or patients with lacking data. None of the recruited patients met the criteria to be evaluated for Zollinger-Ellison syndrome or gastrinoma. There were no available medical records or data regarding *H. pylori* infection before the surgery.

### 2.2. Endoscopy

A single examiner, blinded to drug exposure and symptoms, performed UDEs and carefully examined the gastric remnant and the efferent loop. A mucosa defect larger than 5 mm in diameter and extending into the deeper layers of the gastric wall was defined as ulcer, irrespective of the position in the gastric remnant or anastomotic area. At least 3 biopsies were taken from the gastric remnant, from the anastomosis and efferent side, and from the ulcers. Based on the endoscopic findings, we divided the patients into 2 groups: the study group (patients with ulcer) and the control group (patients without ulcer on UDE). The presence or absence of macroscopic bile pooling was noted on endoscopic intubation of the stomach.

### 2.3. Histology

The biopsies were examined for routine investigation. Biopsy specimens were fixed in 10% buffered formalin, routinely processed, embedded in paraffin, and stained with hematoxylin-eosin, PAS-Alcian blue, and Giemsa. *H. pylori* infection was considered negative if *H. pylori* was absent from all biopsy sites and positive if at least one histology test was positive, including the immunohistochemical study. The degree of mucosal chronic inflammation, activity, *H. pylori* infection, glandular atrophy, and intestinal metaplasia were classified according to the updated Sydney system, but the patients have been assigned according to the presence or absence of changes in the ulcer and nonulcer group. We also evaluated dysplasia according to the modified Vienna classification, but patients with dysplasia or neoplasia were excluded. Patients without important inflammation, but with prominent foveolar hyperplasia, fibromuscular replacement of the lamina propria, and congestion of superficial mucosal capillaries, were diagnosed with reactive gastropathy.

### 2.4. Statistical Analysis

The description of demographic and clinical characteristics was performed using absolute frequencies (number of cases) and relative frequencies (%). The bivariate analysis was realized using Fisher's exact test or chi-square tests. The crude odds ratio and associated 95% confidence interval (CI) were used to quantify the degree of association. An estimated significance level (*p* value) lower than 0.05 was considered statistically significant.

In order to establish the independent predictive factors for the occurrence of gastric stump ulcers, we used the binomial logistic regression as an appropriate statistical method. In the development of model prediction, we selected all variables with a *p* value smaller than 0.25 in the univariate regression analysis, along with all variables of known clinical importance. The presence of multicollinearity in the model was tested using variance inflation factors. The performance and consistency of the final model were assessed by McFadden's (*R*^2^) coefficient, while the classification capacity was evaluated using receiver operating characteristic (ROC) curves and associated areas under the curve (AUC) as a measure. The values of AUC are comprised between 0.5 (no discrimination) and 1.0 (perfect classification), and according to the convention introduced by Hosmer and Lemeshow [16], values above 0.8 indicate an excellent discrimination ability for the model. The estimated AUC was accompanied by 95% associated confidence interval.

The level of statistical significance for all two-tailed tests was set to 0.05.

The advanced environment for statistical computing R (v.3.2.4, Vienna, Austria) was used for statistical analysis.

## 3. Results

### 3.1. Bivariate Analysis

The study was performed in 21 patients with gastric ulcer irrespective of localization (anastomotic area or the rest of the gastric remnant—study group) and 55 controls (nonulcer group). The distribution of demographical and clinical variables of interest (age, sex, smoking, alcohol consumption, *H. pylori* infection, drug consumption, biliary reflux, comorbidities, and symptomatology) is shown in Table 1.

Chi-square or Fisher's exact tests showed that alcohol consumption had a significant positive association with ulcer (*p* = 0.008), which indicated that patients who reported an alcohol consumption (≥10 units/week, 1 unit = 10 ml pure alcohol) were more likely to have a gastric ulcer than nonconsumers. Regarding the habit of smoking (*p* = 0.064) and LDA consumption (*p* = 0.063), there was a tendency toward statistical signification for positive association.

Analyzing the frequency of histological findings in our groups, we found that the proportions of patients with reactive gastropathy were similar in ulcer and nonulcer groups (76.2% vs.70.9%, *p* = 0.645). Gastric atrophy and/or intestinal metaplasia were detected more frequently in patients with ulcer than in the control group, but the difference was not statistically significant (38.1% vs. 27.3%, *p* = 0.358).

In our study, 54 patients (71.05%) had Billroth I anastomosis and 19 patients (25%) had Billroth II anastomosis. The frequency of ulcer development was 90.5% in Billroth I anastomosis and 10.5% in Billroth II anastomosis.

### 3.2. Development of the Prediction Model

The univariable odds ratios, as a measure for the association between each studied predictor and gastric ulcer, are shown in Table 2.

From a total of 14 variables being considered potential predictors for gastric ulcer, 8 variables had *p* < 0.25 and were used for the first model selection. A preliminary main effects model was specified, and it was considered the final model, because we did not find other independent variables with *p* ≥ 0.25 or significant interaction terms.

According to our multivariate regression analysis results (Table 3), the final multivariable ulcer gastric prediction model had the following as predictors: age, gender, smoking, NSAIDs, *H. pylori* infection, alcohol consumption, biliary reflux, chronic kidney diseases, respiratory diseases, and osteoarthritis. Each predictor had a variance inflation factor lower than 1.5, so there was no problem of multicollinearity.

There were two factors, alcohol consumption (OR = 6.68, 95% CI: 1.29–41.14) and the presence of biliary reflux (OR = 6.12, 95% CI: 1.36–38.26), that were significantly associated with increased odds of gastric stump ulcer. The presence of chronic kidney disease was also positively associated with gastric ulcer, with a tendency toward statistical significance (*p* = 0.081).

### 3.3. Model Performance

**Figure 2:**
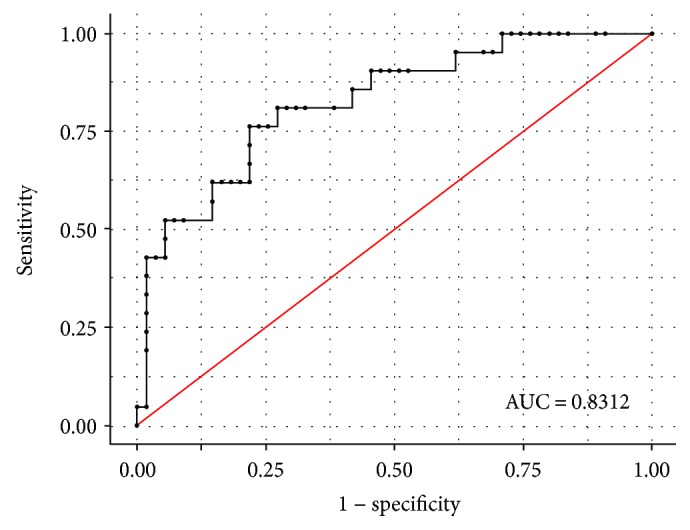
Discrimination ability of the logistic model (ROC curve). Note: the area under the curve demonstrated a good discrimination ability of the logistic model (good capacity of the logistic model to predict ulcer gastric presence correctly). The graph also showed sensitivity values versus 1 − specificity values for different cut-off classification probability values.

The model's McFadden's coefficient (*R*^2^) was 0.390, and the model likelihood ratio test was significant (LR *χ*^2^ = 24, df = 9, *p* = 0.0043). The model also demonstrated an acceptable classification ability (AUC = 0.83, 95% CI: 0.73–0.93) (Figure 2) and a reasonable goodness-of-fit to data (Hosmer-Lemeshow test: *χ*^2^ = 4.76, df = 8, *p* = 0.783).

## 4. Discussion

The potential complications after gastric resection are biliary reflux with consecutive gastropathy/gastritis, ulcers of the gastric remnant, nutritional disturbances, dumping syndrome, and “gastric stump cancers” [17–19]. Ulcers of the gastric stump are widely studied in the medical literature. In consecutively investigated patients, we noticed that the vast majority had been resected for ulcer more than 15 years before and some of them were in the “PPI era.” We do not have a clear explanation, but it may be related to the “doctor's preference” for surgical therapy of ulcer-complicated episode instead of medical or endoscopic therapy in some Romanian hospitals, where medical therapeutic means were not fully available at that time. The most studied were MU, divided according to the time of discovery after surgery into early MU, occurring within 1 to 10 months after surgery [20, 21], and late MU, developing many years after gastrectomy [22]. The etiology of early MU seems to be linked to local tissue injury, the type of suture [22–24], local ischemia, and inflammatory reaction [22]. Nevertheless, the etiology of late MU ulcers is not very well known [12]. Our study questioned clinical and pathological predisposing factors for late recurrent gastric ulcers in patients with long-term gastric surgery for PUD, both in the anastomotic area (MU) and in the rest of the gastric remnant. Surprisingly, none of the known major risk factors for gastric ulcer (gastrotoxic drugs, *H. pylori* infection) remained predictors for recurrent ulcer in patients with previous gastric surgery for PUD in our work, except for alcohol consumption and biliary reflux.

We registered a frequency of ulcers of 27.6% among patients with gastric resection, similar to the data of Chung et al. who detected a prevalence of 27.1% [12]. The present observations suggest an increased frequency of recurrent ulcers in patients with more than 15 years after previous surgery investigated on UDE, as the reported PUD in the endoscopic population was 5.6% in an American study [25] and 12.5% in our endoscopic series (data not published). Despite the known predisposing factors discussed below, a possible individual predisposition or vulnerability of the gastric remnant, involving more subtle unknown environmental or genetic factors that unbalanced the protective and aggressive mechanisms of the gastric mucosa, can explain this observation [26, 27].

The relationship between the surgical reconstruction technique and ulcer formation is debatable according to the available published data. The majority of works suggest that patients with Billroth II anastomosis are more prone to developing endoscopic ulceration than patients with Billroth I, probably due to the increased bile reflux [12]. Another study found no significant difference between the two anastomosis models and ulcer occurrence [23]. Because the contingency table of Billroth I anastomosis, Billroth II anastomosis, and gastric ulcer contained observed frequencies lower than 5, we did not use the type of anastomosis as a factor in multivariate analysis, in order to avoid large standard errors of estimate.

Epidemiological and clinical research established that *H pylori* infection represents a major cause of peptic ulcers and gastric cancer on the normal stomach [2], but its role in developing gastric stump ulcer is still unclear [8, 9]. With a lower frequency (4.8%) in our ulcer group than in the nonulcer group (14.5%), we found no statistically significant association between recurrent gastric ulcer and *H. pylori* infection in the gastric stump. The diminished role of *H. pylori* may be related to the low frequency of infection in the gastric stump. The spontaneous phenomenon of the germ clearance over the years seems to be a consequence of the antibacterial effect of the bile, the increase in gastric pH, or the replacement of normal mucosa with atrophic mucosa [28, 29].

Histological features in gastric mucosa samples (reactive gastropathy, active or inactive gastritis, and premalignant lesions) are not predictive for ulcer risk in resected patients. The most frequent histologic finding in resected patients was reactive gastropathy (72.4%), while inflammatory findings were rare (18.4%). The high frequency of gastric atrophy/intestinal metaplasia associated with a decreased acid secretion in the ulcer group and the low frequency of *H. pylori* infection and inflammation suggest the role of other factors instead of acid secretion or histological features in gastric stump ulcers. Even if *H. pylori*-related ulcer was the probable cause of the first complicated PUD in the majority of our patients, the long-term evolution of the disease, with the clearance of the germ, tends not to offer an efficient protection against recurrent ulcer.

The microenvironment is changing dramatically after gastric resection, especially due to biliary reflux, which becomes a permanent aggressor of the gastric layer, increasing the pH of the gastric content [28, 29]. The effect of chronic biliary reflux is the appearance of reflux gastritis, associated with histological changes. Even if the irritant effect of biliary reflux on the gastric mucosa is well known, its influence on developing gastric stump ulcer is not as well established as its role in gastric stump cancer [10, 29]. In our final regression model, biliary reflux remains a good predictor for ulcer, even if it is studied in the presence of other variables.

The use of NSAIDs and antithrombotic drugs is well known to increase the risk of gastric ulcer two- or three-fold in the general population [1, 2]. It is believed that among patients with gastric resections, the use of the gastrotoxic medication has a similar effect. However, no controlled clinical studies on the consumption of NSAIDs and antiplatelet therapy have been conducted among patients with gastric resection for PUD, in order to determine the risk magnitude in this specific population. In our current research, the use of NSAIDs and LDA in the presence of other variables tended to influence the occurrence of ulcer in the gastric stump, possibly due to the complex interplay of aggressive mechanisms and other factors; their role did not reach statistical significance.

Although smoking or alcohol consumption are not the primary case of PUD, they have been reported as contributors to ulcer pathogenesis in some reports [30–33]. Several studies suggested that smoking and alcohol consumption might increase the risk of developing gastric ulcer among patients with the gastric stump, through different mechanisms. There was an influence of smoking on ulcer occurrence in our study, but not in the presence of other variables. Alcohol consumption (>10 units/week) remained a good predictor for ulcer recurrence in gastric-resected patients. Our observations support the different role of local aggressive factors in the gastric stump compared to the normal stomach, as alcohol consumption is not a major risk factor for gastric ulcer in the general population.

Symptoms were present in both the ulcer and nonulcer groups with comparable frequencies. Most of them can be explained by the presence of biliary reflux, common among patients with gastric resection [28], but the lack of specific premonitory symptoms for gastric stump severe lesions should be emphasized.

Concomitant diseases were not systematically investigated as predisposing factors for recurrent ulcers in patients with gastric surgery. Our previous work suggested the role of cerebrovascular diseases or congestive heart failure as independent risk factors for ulcer in nonresected aspirin consumers [33, 34]. Antithrombotic drug consumption was significantly associated with cardiovascular diseases, and on univariate analysis, antithrombotic drug consumption had the tendency toward statistical significance for ulcer occurrence, while cardiovascular diseases were not (using the chi-square test for the frequency of ischemic cardiac disease (*p* = 0.690) and for the frequency of heart failure (*p* = 0.442) in the studied groups). In the final regression model, we choose as an interest predictor drug consumption, but not cardiovascular diseases, in order to avoid the multicollinearity effect. Our findings suggest a possible influence of respiratory, articular, or renal chronic diseases on ulcer reoccurrence. When they were studied in the presence of other variables, only kidney diseases became a possible predictor for ulcer. Renal function impairment has been proved to increase the general risk for gastric ulcers and bleeding in several studies [34–36]. The possible described mechanisms are abnormal platelet function, mucosal integrity, or acid secretion in uremic patients that can promote local small bleeding and delay ulcer healing [35, 37], and these can also be available in the gastric remnant.

Despite its limitations—a study performed in an endoscopic population, with a relatively low number of cases, with no information on the vagotomy procedure or serological markers of decreased acid secretion—our study revealed the predictive role of the most known risk factors for ulcer development in the gastric stump. Because of the relatively small number of cases compared to the number of factors, the regression model must be retested in future studies in order to highlight the contribution of factors with a tendency toward statistical significance, to improve with another factors (such as the type of anastomosis) or to confirm known factors as *H. pylori* infection, reported to account for the majority of ulcers in our endoscopic population [38, 39].

To the best of our knowledge, there are no recently published reports regarding the interplay between clinical and histological factors in gastric stump ulcers, at more than 15 years after surgery. Our findings can offer a perspective on protective strategy development in gastric-resected patients irrespective of the cause and suggest that certain predisposing factors for gastric stump ulcer can be influenced.

## 5. Conclusions

Biliary reflux and alcohol consumption, but not *Helicobacter pylori* infection or gastrotoxic drug consumption, are the most important predictors for ulcer recurrence in patients with gastric surgery for peptic ulcer disease after more than 15 years.

## Figures and Tables

**Table 1:** The distribution of independent variables of interest in the studied groups.

Variables	Nonulcer group (*n* = 55)	Ulcer group (*n* = 21)	*p* value^a^
	No. of cases (%)	No. of cases (%)	
*Gender*			
Female	18 (32.7)	6 (28.6)	0.789
Male	37 (67.3)	15 (71.4)	
*Age*			
<60 years	15 (27.3)	7 (33.3)	0.778
≥60 years	40 (72.7)	14 (66.7)	
*Smoking*			
No	46 (83.6)	13 (61.9)	0.064
Yes^b^	9 (16.4)	8 (38.1)	
*LDA* ^c^			
No	34 (61.8)	8 (38.1)	0.063
Yes	21 (38.2)	13 (61.9)	
*Clopidrogrelum*			
No	47 (85.5)	16 (76.2)	0.338
Yes	8 (14.5)	5 (23.8)	
*NSAIDs* ^d^			
No	51 (92.7)	17 (81.0)	0.135
Yes	4 (7.3)	4 (19.0)	
*Anticoagulants*			
No	49 (89.1)	17 (81.0)	0.449
Yes	6 (10.9)	4 (19.0)	
*H. pylori*			
Negative	47 (85.5)	20 (95.2)	0.430
Positive	8 (14.5)	1 (4.8)	
*Alcohol consumption* ^e^			
No	46 (83.6)	11 (52.4)	0.008
Yes	9 (16.4)	10 (47.6)	
*Biliary reflux*			
No	23 (41.8)	4 (19.0)	0.106
Yes	32 (58.2)	17 (81.0)	
*Diabetes mellitus*			
No	42 (76.4)	15 (71.4)	0.657
Yes	13 (23.6)	6 (28.6)	
*Chronic kidney disease*			
No	42 (76.4)	12 (57.1)	0.156
Yes	13 (23.6)	9 (42.9)	
*Chronic liver disease*			
No	33 (60.0)	10 (47.6)	0.439
Yes	22 (40.0)	11 (52.4)	
*Respiratory disease*			
No	44 (80.0)	13 (61.9)	0.139
Yes	11 (20.0)	8 (38.1)	
*Osteoarthritis*			
No	42 (76.4)	13 (61.9)	0.255
Yes	13 (23.6)	8 (38.1)	
*Symptoms* ^f^			
No	21 (38.2)	9 (42.9)	0.795
Yes	34 (61.8)	12 (57.1)	

^a^Obtained from chi-square or Fisher's exact test; ^b^over 5 cigarettes/day including quitters during the past 5 years; ^c^LDA = low-dose aspirin (75–125 mg/day); ^d^NSAIDs: nonsteroidal anti-inflammatory drugs, regular daily doses; ^e^more than 10 units/week, 1 unit = 10 ml pure alcohol; ^f^symptoms = at least one symptom from upper abdominal pain, heartburn, and nausea/vomiting.

**Table 2:** Univariable odds ratio for considered independent variables of gastric ulcer.

Variables	*p* value^‡^	OR_crude_	95% CI [lower limit, upper limit]
*Independent variables*			
Smoking^a^	0.048^∗^	3.15	[1.01, 9.93]
LDA^b^ (no)	0.067^∗∗^	2.63	[0.95, 7.68]
Clopidrogrelum (no)	0.341	1.84	[0.49, 6.35]
NSAIDs^c^ (no)	0.149^∗∗∗^	3.00	[0.65, 13.99]
Anticoagulants (no)	0.353	1.92	[0.45, 7.57]
*H. pylori* (negative)	0.262	0.29	[0.02, 1.76]
Alcohol consumption^d^	0.007^∗^	4.65	[1.54, 14.60]
Biliary reflux (absent)	0.071^∗∗^	3.05	[0.98, 11.69]
Diabetes mellitus (absent)	0.657	1.29	[0.40, 3.93]
Chronic kidney disease (absent)	0.103^∗∗∗^	2.42	[0.83, 7.09]
Chronic liver disease (absent)	0.332	1.65	[0.60, 4.61]
Respiratory disease (absent)	0.109^∗∗∗^	2.46	[0.81, 7.45]
Osteoarthritis (absent)	0.211^∗∗∗^	1.99	[0.66, 5.85]
Symptoms^e^	0.709	0.82	[0.30, 2.33]
*Controls*			
Gender (female)	0.728	1.22	[0.42, 3.88]
Age (<60 years)	0.603	0.75	[0.26, 2.30]

Note: the reference category for each variable was written in parenthesis. ^‡^Estimated significance level obtained from Wald's test; ^∗^*p* < 0.05, ^∗∗^*p* < 0.10, ^∗∗∗^*p* < 0.25. ^a^<5 cigarettes/day, including quitters during the past 5 years; ^b^LDA = low-dose aspirin (75–125 mg/day); ^c^NSAIDs: nonsteroidal anti-inflammatory drugs, regular daily doses; ^d^less than 10 units/week, 1 unit = 10 ml pure alcohol; ^e^symptoms = the absence of any symptom from upper abdominal pain, heartburn, and nausea/vomiting; OR = odds ratio; CI = confidence interval.

**Table 3:** Multivariable odds ratio from the final logistic model of gastric ulcer.

Variables	OR_adj_	95% CI [lower limit, upper limit]	*p* value^‡^
*Independent variables*			
NSAIDs (no)	4.76	[0.55, 43.65]	0.132
*H. pylori* (negative)	0.35	[0.02, 3.18]	0.387
Alcohol consumption (<10 U/week)	6.68	[1.29, 41.14]	0.013
Biliary reflux (absent)	6.12	[1.36, 38.26]	0.026
Chronic kidney disease (absent)	3.45	[0.84, 15.13]	0.081
Respiratory disease (absent)	1.79	[0.33, 9.43]	0.405
Osteoarthritis (absent)	2.69	[0.51, 14.73]	0.242
*Controls*			
Gender (female)	1.08	[0.22, 5.48]	0.911
Age (<60 years)	0.59	[0.14, 2.58]	0.425

Note: the reference category for each variable was written in parenthesis; OR_adj_ = adjusted OR; ^‡^estimated significance level obtained from Wald's test; NSAIDs: nonsteroidal anti-inflammatory drugs; CI = confidence interval.
